# Bolstering Cognitive and Locomotor Function in Post-Stroke Dementia Using Human–Robotic Interactive Gait Training

**DOI:** 10.3390/jcm12175661

**Published:** 2023-08-31

**Authors:** Yunhwan Kim, Chanhee Park, Buhyun Yoon, Joshua (Sung) H. You

**Affiliations:** Sports Movement Artificial Robotics Technology (SMART) Institute, Department of Physical Therapy, Yonsei University, Wonju 26943, Republic of Korea; aidenkim@yonsei.ac.kr (Y.K.); chaneesm@gmail.com (C.P.); ybh54888@naver.com (B.Y.)

**Keywords:** cognition, neurorehabilitation, post-stroke dementia, robotic rehabilitation

## Abstract

Studies have reported inconclusive results regarding the effectiveness and clinical indications of the exclusive use of human–robotic interactive gait training (HIT) in patients with post-stroke dementia (PSD). This study aimed to compare the effects of human–robotic interactive gait training (HIT) and conventional physiotherapy (CPT) on cognitive and sensorimotor functions, trunk balance and coordination, dynamic and static balance, and activities related to daily living performance in patients with PSD. Forty-eight patients with PSD who received 60-minute therapy sessions three times per week for 6 weeks were assigned to either the CPT (*n* = 25) or HIT (*n* = 23) group. The clinical outcomes included the scores of the mini-mental state examination (MMSE), Fugl–Meyer assessment (FMA), trunk impairment scale (TIS), Berg balance scale (BBS), and modified Barthel index (MBI). Friedman tests were conducted at *p* < 0.05. The Friedman tests showed that HIT had superior effects to CPT in relation to MMSE, FMA, and TIS (*p* < 0.05), but not in relation to BBS and MBI (*p* > 0.05). Our results provide promising clinical evidence that HIT significantly improves cognitive and sensorimotor recovery functions, as well as trunk balance and coordination, in patients with PSD who cannot concurrently perform dual cognitive–locomotor tasks.

## 1. Introduction

Post-stroke dementia (PSD) is a common cognitive and locomotor impairment following cerebrovascular microcirculation damage [1]. The risk of developing dementia doubles in patients with stroke, with an incidence rate of approximately 30% [2]. Primary cognitive impairments include orientation, memory, and attention; Refs. [3,4] comorbid motor impairments include declines in sensorimotor function, trunk balance and coordination, dynamic and static balance, and the performance of activities of daily living (ADL) [5]. In addition, patients with PSD, who have limited cognitive status and attention, may find it challenging to concurrently perform dual cognitive–locomotor tasks owing to the misallocated attentional resources [6]. Locomotor control is a subcortical or reflexively mediated rhythmic locomotor function generated by spinal cord central pattern networks [7,8], as well as supraspinal regulation from cerebrocerebellar, cortico-reticular, and vestibulospinal connections [9]. It is modulated via cortical motor commands if high-level cognitive–locomotor processes are involved in obstacle navigation to avoid tripping or falling. Unfortunately, gait function is often compromised in most patients with PSD, often resulting in fatal falls [10].

To mitigate cognitive and locomotor impairments, task-oriented body weight-supported treadmill training (BWSTT) and robotic-assisted gait training (RAGT) have been utilized in patients with stroke, though studies have produced variable outcomes [11,12,13]. Duncan et al. (2011) demonstrated that BWSTT enhanced walking mobility (8%) and balance (15%) in 408 patients with chronic hemiparetic stroke compared to the controls, who received a home-exercise program; however, no cognitive–locomotor recovery outcome measure was obtained [11]. The BWSTT technique only involved intensive and repetitive treadmill-based stepping training, without considering the cognitive element. Similarly, one RAGT case study reported the positive effects of RAGT on cognition and motor function (balance), with an improvement of 29% and 25% in the mini-mental state examination (MMSE) and Tinetti tests [13]. RAGT, in the previous study, included only the hip joint- and knee joint-assisted treadmill-based robotic training element, rather than the cognitive element; hence, it was difficult to determine the effectiveness and underlying rationale of RAGT on cognitive function (as evidenced by MMSE), including time orientation, memory, and attention.

A clear need exists to effectively and sustainably restore cognitive and locomotor impairments during stroke rehabilitation, especially in patients with PSD, to reduce the risk of falls and improve independent ADL performance [14]. Recently, we developed and designed the Walkbot human–robotic interactive gait training (HIT) (P&S Mechanics, Seoul, Republic of Korea) program to facilitate cognitive and locomotor function in patients with PSD by providing ample task-specific training intensity (repetition) for engram and neuroplasticity [15]. In addition, this system customizes impedance-based controllers to achieve the desired joint trajectories, utilizing user capability-dependent robotic-assisted guidance [15]. Unlike the previously utilized BWSTT and RAGT, our HIT enables clinicians concurrently to provide a combination of cognitive and locomotor elements of the recovery regimen. Specifically, the cognitive training element involved verbal cognitive task interaction based on activities of daily living, including orientation (e.g., present time—morning, noon, and evening, as well as location), attention (e.g., counting numbers 1–10 to engage in the cognitive–locomotor stepping task), and memory (e.g., recalling meals, age, and family information). On the other hand, the locomotor training element entailed the real-time kinematic and kinetic data results, including those from the active lower-limb exoskeletons. Our previous Walkbot RAGT studies [15,16,17,18,19,20,21] provided promising clinical implications and significance, contributing to the improvement of the rehabilitation balance and locomotor recovery outcomes and quality of life in patients with stroke, which provides a stronger justification and rationale for selecting HIT over the existing BWSTT and RAGT interventions. Jeong et al. (2021) reported favorable enhancements in MMSE cognitive function (14.6%) and FMA sensorimotor function (14.6%) based on the effectiveness of Walkbot RAGT in 218 individuals with hemiplegic stroke [16]. Park et al. (2020) reported that the Walkbot ankle–knee–hip interlimb coordinated locomotor training improved kinematics (angle: hip joint 9.0%; knee joint: 19.0%), kinetics (active force: hip joint 55.3%; knee joint: 97.0%; ankle joint: 69.7%) in 20 individuals with subacute stroke compared to the controls, who received conventional physical therapy [17]. Park et al. (2020) showed more substantial improvements in ambulation (34.7%); cardiopulmonary function (13.9%); depression (58.0%); fear of falling (55.9%), as measured by the functional ambulation scale; heart rate; Beck depression inventory-II; and activity-specific balance confidence, following the application of the Walkbot RAGT, in individuals with acute hemiplegia than in controls [17]. Indeed, this collective evidence supports advantageous clinical benefits and ramifications in cognitive, locomotor, and psychological outcomes after the application of Walkbot RAGT. However, despite the important clinical ramifications of HIT in PSD, its therapeutic effects on cognition and locomotor function remain unknown. Therefore, this study aimed to compare the effects of HIT and CPT on cognitive function, sensorimotor function, trunk balance and coordination, dynamic and static balance, and ADL performance, as assessed via MMSE, FMA, the trunk impairment scale (TIS), BBS, and MBI, respectively, in patients with PSD. We hypothesized that HIT would produce superior effects to conventional physiotherapy (CPT) in terms of cognitive function, sensorimotor function, trunk balance and coordination, dynamic and static balance, and ADL performance in patients with PSD.

## 2. Materials and Methods

### 2.1. Participants

This retrospective clinical study included patients treated with HIT and CPT as inpatients in a major rehabilitation hospital in Seoul. A convenience sample of 48 patients with PSD (mean age = 68.25; 19 women) admitted from July 2019 to December 2021 was evaluated, and the data were stored in the Clinical Data Warehouse at the Rehabilitation Hospital (Seoul, Republic of Korea), including a database of electronic medical records obtained from inpatients to perform further analysis.

Inclusion criteria were as follows: (1) stroke onset within 1 to 12 months; (2) patients diagnosed with dementia after a stroke [22]; (3) MMSE scores in the range 10–25 for mild and moderate dementia [23]; (4) age: 18–99 years old; (5) height: 132–200 cm; (6) the ability to ambulate at least one step with a device/assistance (functional ambulation categories = 1); (7) hip–knee joint length of 33 to 48 cm; (8) knee–ankle joint length of 33 to 48 cm; (8) can follow instructions and engage in verbal communication. Exclusion criteria were as follows: (1) body weight > 135 kg; (2) stage 2 uncontrolled hypertension with blood pressure > 160/100 mmHg; (3) cardiopulmonary impairments affecting the ambulation test; (4) integumentary impairment, such as skin breakdown or bedsores around the suspension belt application area; (5) significant and persistent mood disorders (depression, anxiety, and emotional lability), attention disorder, and delirium [24,25]; (6) lower extremity fixed contracture or deformity; (7) bone instability (non-consolidated fractures, unstable spinal column, or severe osteoporosis requiring treatment with bisphosphonates); (8) other neurodegenerative disorders (amyotrophic lateral sclerosis and Parkinson’s disease); (9) significant pain and sensory deficit; (10) aphasia and dysarthria causing communication problems.

### 2.2. Study Design

**Figure 1:**
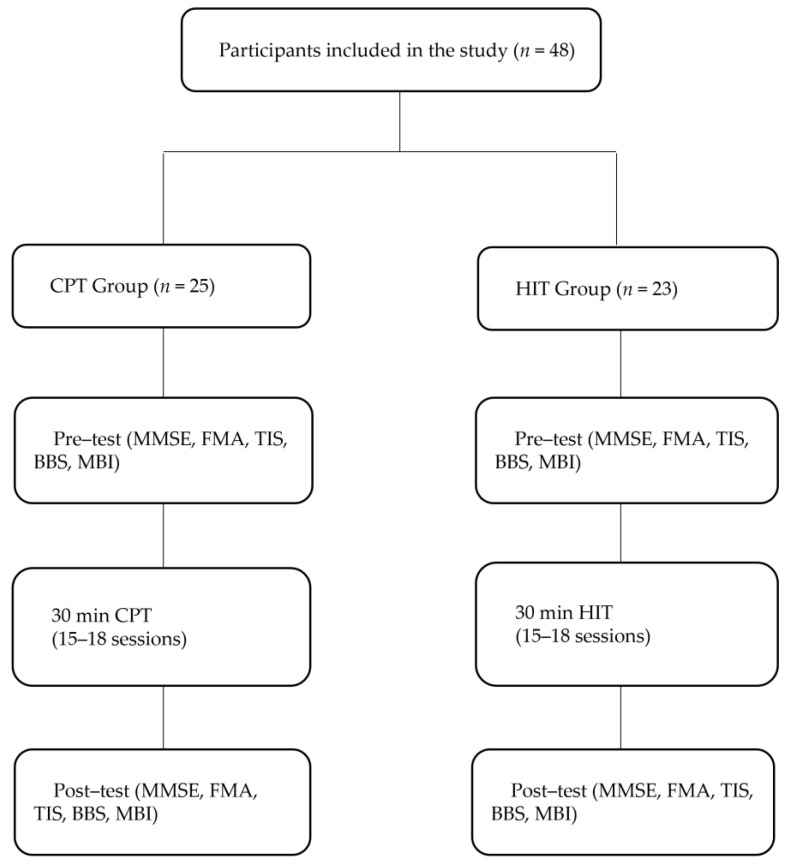
Flow chart.

This retrospective study research compared HIT and CAT on the basis of cognitive–locomotor control in patients with PSD. All patients underwent one of the rehabilitation protocols (CPT or HIT), which consisted of 60-min sessions 3 times/week for 6 weeks. CPT and HIT were delivered by four physical therapists; two robotic-certified and two neurodevelopmental treatment (NDT)-certified therapists. The CPT group received the usual inpatient care of one 30-min physical therapy session per day and an additional 30 min of a standard therapy session focused on pre-gait and/or gait training activities. The HIT group received the usual inpatient therapy of one 30-min physical therapy session and an additional 30 min of HIT session. This study attempted to standardize the intervention protocol, including physical therapy, occupational therapy, and speech–language therapy between the groups. Assessments were conducted by the same evaluator, who was blinded to the group assignments and results. The flowchart of this study is shown in Figure 1.

The CPT was based on the NDT framework and collective clinical evidence of therapeutic exercises [26]. The NDT-trained physical therapist provided the intervention required to inhibit abnormal movement and tone and facilitate normal postural tone and coordinated movements during therapeutic exercises to improve cognitive and locomotor function [26]. Therapeutic exercises included core stabilization, NDT sequence-based functional training (supine, side-lying, hook-lying, bridging, prone on elbow, quadruped, sitting, kneeling and half-kneeling, and standing), gait-specific exercises, passive stretching and strengthening exercises for mobility and stability, and dynamic or static balance exercises, which gradually progressed to overground gait training with or without the use of assistive devices (cane or walker) [27,28]. The corresponding interventions included ROM exercises, stretching, strengthening, and treadmill training to improve gait function. These interventions are illustrated in detail in Supplementary File S1. Once the patient had progressed to standing, they engaged in 20–30 min of BWSTT.

**Figure 2:**
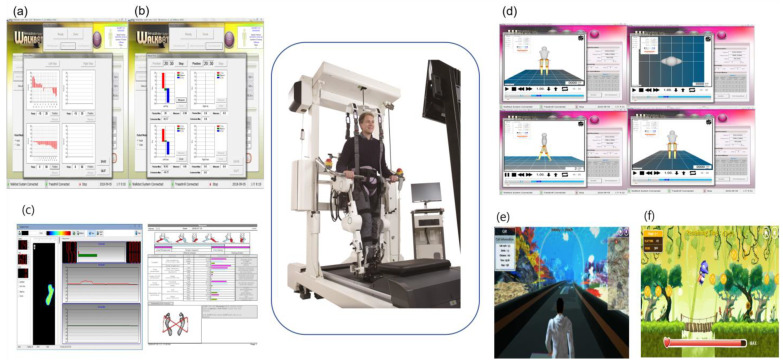
Walkbot human−robotic interactive gait training system (**a**): active joint angular displacement feedback; (**b**) active force/torque feedback; (**c**): weight-bearing center of pressure; (**d**): gait kinematics and kinetics in ankle−knee−hip joints; (**e**): augmented reality; (**f**) virtual reality.

The HIT utilized Walkbot (P&S Mechanics, Seoul, Republic of Korea), which is a human–robotic interactive lower limb exoskeleton with powered ankle, knee, and hip joints, to provide optimal and coordinated ankle–knee–hip gait kinematics and kinetics during locomotor retraining. The details of the Walkbot system (biomechanical measurements for kinematics and kinetics) are included in Supplementary File S2. The anthropometric data of each patient, including weight, height, thigh, shank, ankle height, and foot size, were measured and entered into the patient database. These values were subsequently used to adjust the exoskeleton leg length and optimal gait cycle based on each patient’s condition. A suspension vest was used to secure the chest and pelvic girdle with elastic straps, which were connected to the harness mounted on the counterweight system to sustain body weight during the HIT. The patient’s initial clinical condition determined the body weight to be sustained (approximately 40–60% of the total) in the first session, which was thereafter decreased by 5–10% per session [21]. Stride length was initially set at 1.0–1.6 m/cycle, and walking velocity was set at 1.00–1.20 km/h. The walking velocity was consecutively gradually increased by 0.1 km/h every 5 min, as tolerated, up to 2.40–2.60 km/h (maximally adjustable to 3.00 km/h) [21]. The guidance force mode in the HIT system was used to accurately increase the active engagement during robotic-assisted gait retraining. As the patient’s walking ability improved from the initial target level (e.g., 40 Nm), the Walkbot system interactively adjusted the walking speed and resistive torque parameters while attempting to minimize the kinematic trajectory errors. The assistance guidance force was systematically reduced from 100 (passive mode) to 0% (active mode) by compensating for the weight, resistance, and inertia to accomplish symmetrical optimal gait patterns; the differential mode could be applied to the more affected (hemiparetic) leg. Furthermore, there was real-time audiovisual biofeedback regarding the gait kinematics (joint angles) and kinetic forces (active, resistive torque, and stiffness) from the ankle, knee, and hip interlimb joint movements [17]. The patients were continuously encouraged during and after each session based on the real-time kinematic and kinetic data results, including those from the active lower-limb exoskeletons. The guidance force, speed, and body weight support were systematically adjusted during each session according to the patient’s abilities to maximize their training intensity while remaining motivated through enjoyable, verbal, and cognitive task interactions based on activities of daily living, including orientation (e.g., present time—morning, noon, and evening, as well as location), attention (e.g., counting numbers 1–10 to engage in the cognitive–locomotor stepping task), and memory (e.g., recalling meals, age, and family information) [16]. The therapist ensured accurate robotic locomotor training strategies by monitoring the real-time kinematic (e.g., spatiotemporal parameters and ankle, knee, and hip joint angles) and kinetic gait trajectories (ankle, knee, and hip joint active and resistive forces and torques) displayed on the Walkbot HIT system monitor (Figure 2).

### 2.3. Clinical Outcome Measurements

In this study, standardized tests encompassing MMSE, FMA, TIS, BBS, and MBI were conducted before and after the intervention.

#### 2.3.1. Mini-Mental State Examination

MMSE is a questionnaire extensively used in clinical and research settings to measure the outcomes of cognitive function. MMSE includes simple questions grouped into the sub-sections of orientation, registration, attention, and calculation. All of the scores are summed to obtain a final score of 30. The reported MMSE scores are 30–26 (normal), 25–20 (mild), 19–10 (moderate), and 9–0 (severe) for dementia [23]. The reliability (intraclass correlation coefficient; ICC = 0.92) and validity (*r* = 0.78) of the outcome measures have previously been established [29,30].

#### 2.3.2. Fugl–Meyer Assessment

The FMA is commonly used to assess sensorimotor impairment in patients with hemiparetic stroke, including sensation, motor function, balance, joint range of motion, and pain. The FMA scale is an ordinal scale with three points for each item and ranges from 0 (‘cannot perform’) to 2 (‘performs completely’). The reflex activity item is measured using a total of two points, with a score of 0 (‘cannot perform’) or 2 (‘performs completely’) in the absence or presence of a reflex, respectively [31]. The maximum total score for the FMA is 226, although it is typical to assess all domains separately, as required. The reliability and validity of the outcome measurement tests were reported to be ICC = 0.97 and *r* = 0.98, respectively [32].

#### 2.3.3. Trunk Impairment Scale

The TIS assesses the motor impairment of static and dynamic sitting balance and coordination of trunk movements. Scores range from 0 (‘minimal performance’) to 23 (‘perfect performance’) [33]. The three subscales, i.e., static sitting balance (0–7), dynamic sitting balance (0–10), and coordination (0–6), were rated by a certified physical therapist. The intra- and inter-observer reliabilities of TIS score in stroke patients are reported to be high, as ICC = 0.96 and ICC = 0.99, respectively [33,34].

#### 2.3.4. Berg Balance Scale

The BBS was used to assess the performance-oriented balance. The test consists of 14 balance-related tasks ranging from sitting to standing on one foot. The scores range from 0 (‘unable to perform’) to 4 (‘able to perform independently’), with a maximum score of 56 [35]. The inter- and intra-rater reliabilities of the outcome measurements were reported to be ICC = 0.97 and ICC = 0.98, respectively [35].

#### 2.3.5. Modified Barthel Index

The MBI was used to assess the patients’ ADL performances. The MBI is a 10-item functional performance test that evaluates self-care, continence, and locomotion [36]. The values assigned to each item are based on the amount of physical assistance required to perform a task and add up to a total score ranging from 0 (‘entirely dependent’) to 100 (‘fully independent’), with higher scores indicating better levels of physical functioning [37]. The internal consistency reliability coefficient for MBI was *r* = 0.90 [36].

### 2.4. Statistical Analysis

Statistical data were expressed as means and standard deviations. One of the independent *t*-test, Mann–Whitney *U* test, or chi-square test was used to compare the baseline clinical characteristics and demographic data between the CPT and HIT groups. Box’s *M* test was used to determine the assumption of the homogeneity of the dependent variables between groups. A power analysis was conducted using G-Power software (version 3.1.9.4; Franz Faul, University of Kiel, Germany) to assess the minimum sample size requirement based on previous studies. The required sample size was determined to be 48, and power (1 − *β* = 0.8) was based on the effect size (eta squared, η^2^ = 0.6) and the BBS and MBI [17,20]. The Friedman test was used to determine any intervention-related significance in the MMSE, FMA, TIS, BBS, and TIS between groups. If a significant difference was observed, the Mann–Whitney *U* test or Wilcoxon signed-rank post hoc test was used. SPSS for Windows (version 26.0, SPSS, Chicago, IL, USA) was used to conduct these statistical analyses. The *p*-value was set at 0.05.

The Friedman test was used to compare the effects of the interventions. If a significant difference was observed, the Mann–Whitney *U* test or Wilcoxon signed-rank post hoc test was used.

## 3. Results

### 3.1. Demographic Characteristics of Participants

All patients who completed the pre-test, intervention (a minimum of 15 of 18 sessions), and post-test were included in the analysis. Table 1 summarizes the patients’ demographic and clinical characteristics. There were no significant differences in the baseline age, height, weight, onset time, sex, type of stroke distribution, MMSE, FMA, TIS, and BBS variables between the CPT and HIT groups, though there was a significant different between MBI values (Table 1). In addition, Box’s M test showed no significant differences in the baseline between the CPT and HIT (Table 2). No safety issues were reported, and none of the patients experienced any side effects associated with CPT or HIT.

### 3.2. Clinical Outcome Measurements

#### 3.2.1. Mini-Mental State Examination

**Figure 3:**
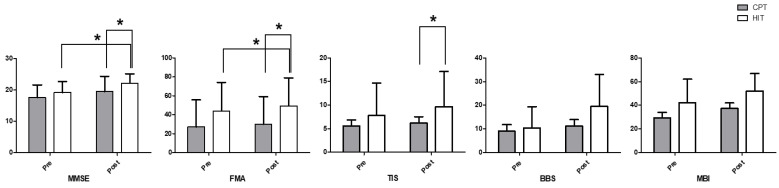
Outcomes of the intervention. * *p* < 0.05.

The Friedman test showed significant differences between the MMSE outcomes (*p* = 0.007) (Table 3). In addition, post hoc analysis confirmed a relatively greater improvement in the MMSE cognitive function after treatment in the HIT group than in the CPT group (Figure 3).

#### 3.2.2. Fugl–Meyer Assessment

The Friedman test showed significant differences between the FMA outcomes (*p* = 0.005) (Table 3). In addition, post hoc analysis indicated a relatively greater increase in FMA sensorimotor recovery function after HIT than after CPT (Figure 3).

#### 3.2.3. Trunk Impairment Scale

The Friedman test showed significant differences between the TIS outcomes (*p* = 0.005) (Table 3). Post hoc analysis confirmed a relatively greater increase in TIS trunk balance and coordination after HIT than after CPT (Figure 3).

#### 3.2.4. Berg Balance Scale

The Friedman test showed no significant differences between BBS outcomes (*p* = 0.116) (Table 3).

#### 3.2.5. Modified Barthel Index

The Friedman test showed no significant differences between MBI outcomes (*p* = 0.085) (Table 3).

## 4. Discussion

In this study, we investigated and compared the effects of HIT and CPT on cognitive and sensorimotor function, trunk balance and coordination, dynamic and static balance, and ADL performance in patients with PSD. As anticipated, HIT enhanced cognitive and sensorimotor recovery functions, trunk balance, and coordination more effectively than CPT. Particularly, HIT substantially improved cognitive function, including orientation, registration, attention, and calculation, contributing to clinically meaningful outcomes in PSD rehabilitation. Unfortunately, the lack of clinical evidence of RAGT in the current literature makes it difficult to compare our results.

Clinical cognitive function analyses revealed greater improvements in the MMSE scores in the HIT group than in the CPT group. This finding paralleled a recent RAGT case study that demonstrated greater improvements in cognitive function measures (MMSE score 29.12%) in a single patient with dementia following 4 weeks of RAGT [13]. Such clinical improvements in cognitive function after HIT and walking aerobic exercise may have resulted from exercise-induced neuroplastic changes in the cognitive domain of the brain. Erickson et al. reported that older individuals with higher levels of aerobic fitness had a greater volume of the anterior hippocampus (5.40%), as measured via functional magnetic resonance imaging (fMRI), and better spatial memory performance [38]. Ten Brinke et al. also showed that aerobic walking exercises significantly improved the left hippocampal volume (5.60%) in patients with mild cognitive impairments, which was associated with better verbal memory [39]. Similarly, MRI evidence demonstrated that 1 year of aerobic walking training enhanced blood flow to the brain and the hippocampus. This outcome may have been due to the increased levels of brain-derived neurotrophic factor, which stimulates neurogenesis in healthy older adults [40].

Our novel HIT approach aims to improve the integration of cognitive and sensorimotor information, which leads to dual cognitive (attention, orientation, memory, and calculation) and locomotor functional recovery by concurrently facilitating detour neural connectivity (‘unmasking’) in the spared corticospinal and subcortical locomotor neural substrates and associated cognitive neural pathways [41,42]. Specifically, HIT may have stimulated the cortico-subcortical locomotor circuits by activating explicit and implicit motor functions caused by specific kinematic and kinetic (proprioceptive or kinesthetic sense) and repetitive recalling of the sensorimotor movement feedback via the Walkbot system [15]. The explicit locomotor training involved in utilizing augmented sensorimotor feedback from a human therapist and robotics may activate corticospinal circuits and associated cognitive pathways (the medial intraparietal area and parietal interconnections with relevant premotor areas of the frontal lobe) [43]. Conversely, the implicit locomotor training was possibly elicited by the repetitive and rhythmic treadmill-based walking stimulating the alternate neuronal connections and connecting the potentially affected corticospinal pathways via the spared subcortical–spinal (central pattern generators) circuits, such as reticulospinal and propriospinal tracts [44]. Our previous functional near-infrared spectroscopy (fNIRS) study demonstrated that the end-effector RAGT has notably facilitated cortical reorganization and activation associated with cognitive–locomotor function, as evidenced by increased oxyhemoglobin levels in the pre-frontal cortex (90.32%) in individuals who experienced chronic hemiparetic stroke [18]. Though functional improvements in integrating cognitive and sensorimotor neuronal substrates have been associated with cognitive and locomotor recovery in PSD patients [41], the precise neuronal control mechanisms and pathways underlying such enhancement remain elusive.

The FMA data analysis also demonstrated a superior and positive effect of HIT on sensorimotor improvement compared to CPT. This result supports those of previous studies that examined the sensorimotor therapeutic effects of RAGT in patients with hemiparetic stroke [45,46,47]. Similarly, Yokota et al. reported that four weeks of RAGT improved sensorimotor recovery function more effectively than CPT in patients with hemiparetic stroke [46]. In addition, You et al. demonstrated substantial improvements in blood oxygenation level-dependent signals in the sensorimotor cortex in patients with stroke after four weeks of locomotor training, supporting the theoretical notion of locomotor training-induced cortical reorganization [47]. Similarly, a recent fNIRS imaging study of patients with hemiparetic stroke showed that the end-effector RAGT improved cortical activity, as evidenced by increased oxyhemoglobin levels in the sensorimotor cortex (84.62%), supplementary motor area (87.28%), and pre-motor cortex (173.82%) [45].

TIS data analysis demonstrated enhanced trunk balance and coordination in HIT compared to CPT. This finding is consistent with a previous RAGT study in patients recovering from a hemiparetic stroke, thus showing greater TIS improvement (12.75%) [18]. Trunk balance and coordination improvement presumably occur because the robotic-assisted locomotor training system provides trunk stabilization, coordinated interlimb ankle–knee–hip joint locomotor movement guidance, and associated proprioceptive and somatosensory feedback [17]. Additionally, the afferent proprioceptive signals may stimulate the spinal cord central pattern generators, thereby facilitating the ascending neuronal locomotor network and training-induced neuroplasticity in the sensorimotor cortex, which, consequently, regulates trunk balance and coordination during locomotion [19,20].

This study had a few limitations. Primarily, as the present study involved a retrospective design, the randomization, blinding, pure control group, and follow-up data are not available, and this issue warrants future investigation. A careful interpretation of our data should be performed when generalizing our findings to a broader population. A future study would require a more comprehensive cognitive intervention protocol, as delineated elsewhere, such as a dual cognitive–locomotor task protocol [48,49]. Robotic-induced cognitive neuroplasticity was not evaluated due to the limitation of current neuroimaging techniques, such as fMRI and functional NIRS. Although fMRI and functional NIRS have superior spatial resolutions, their imaging signals are influenced by locomotor movement and robotic machine electromagnetic artifacts [45]. Nevertheless, our clinical cognitive outcome measure confirmed the proposed benefits of robot-assisted intervention in PSD. Additionally, our study was limited by the lack of a follow-up evaluation, which may have provided practical information about the long-term effects of HIT in PSD, considering its progressive nature. Lastly, for the purpose of meticulous cognitive evaluation, it is recommended that future investigations incorporate supplementary quantitative measurements, such as the frontal assessment battery and the behavioral inattention test.

## 5. Conclusions

Our novel study demonstrated that HIT was more effective than CPT in terms of improving cognitive and sensorimotor function and trunk balance and coordination in patients recovering from PSD. These results provide clinical and evidence-based insights into the utilization of HIT in PSD rehabilitation to maximize recovery of cognitive–locomotor control in patients with PSD.

## Figures and Tables

**Table 1:** Demographic and clinical characteristics of participating patients (*n* = 48).

	CPT ^1^ Group (*n* = 25)	HIT ^2^ Group (*n* = 23)	*p*-Value
Demographic characteristics			
Age (years)	68.16 ± 12.76	68.35 ± 11.09	0.948
Height (cm)	164.60 ± 6.71	164.83 ± 7.80	0.902
Weight (kg)	60.08 ± 10.05	60.92 ± 7.60	0.724
Onset time (months)			
0–3 (%)	15 (60)	13 (57)	0.799
4–6 (%)	7 (28)	6 (26)	
7–12 (%)	3 (12)	4 (17)	
Sex			
Male	16 (64)	13 (57)	0.497
Female	9 (36)	10 (43)	
Type of stroke			
Hemorrhage (%)	12 (48)	15 (65)	0.330
Infarction (%)	13 (52)	8 (35)	
Affected side			
Left (%)	10 (40)	10 (43)	0.809
Right (%)	15 (60)	13 (57)	
Location of lesion			
ACA (%)	2	2	0.446
MCA (%)	17	15	
PCA (%)	4	6	
VB (%)	1	2	
Etc. (%)	1	0	
Clinical characteristics			
MMSE ^3^	18.00	20.00	0.358
FMA ^4^	36.00	49.00	0.156
TIS ^5^	5.00	6.00	0.557
BBS ^6^	5.00	6.00	0.587
MBI ^7^	31.00	36.00	0.154

^1^ CPT, conventional physical therapy; ^2^ HIT, human–robotic interactive gait training, ^3^ MMSE, mini-mental state evaluation; ^4^ FMA, Fugl–Meyer assessment, ^5^ TIS, trunk impairment scale; ^6^ BBS, Berg balance scale, ^7^ MBI, modified Barthel index.

**Table 2:** Box’s *M* test results.

Box’s *M*	85.206
*F*	0.868
*df*_1_	66
*df*_2_	3804.394
Sig.	0.769

**Table 3:** Post-intervention outcome analysis.

	CPT ^1^		HIT ^2^			
	Pre-Test	Post-Test	Pre-Test	Post-Test	χ^2^	*p*-Value
MMSE ^3^	18.00	19.00	20.00	22.00	16.07	0.007 *
FMA ^4^	36.00	40.00	49.00	50.00	16.55	0.005 *
TIS ^5^	5.00	6.00	6.00	10.00	14.27	0.014 *
BBS ^6^	5.00	7.00	6.00	9.00	8.83	0.116
MBI ^7^	31.00	40.00	36.00	54.00	9.66	0.085

^1^ CPT, conventional physical therapy; ^2^ HIT, human–robotic interactive gait training, ^3^ MMSE, mini-mental state evaluation; ^4^ FMA, Fugl–Meyer assessment, ^5^ TIS, trunk impairment scale; ^6^ BBS, Berg balance scale, ^7^ MBI, modified Barthel index; Friedman test * *p* < 0.05.

## Data Availability

The data presented in this study are available on request from the corresponding author.

## Supplementary Materials

The following supporting information can be downloaded at: https://www.mdpi.com/article/10.3390/jcm12175661/s1, File S1: Conventional physiotherapy: it contains details of the three intervention sessions used; File S2: Walkbot human–robotic interactive gait training system: It explains the software sys-tems integrated into the Walkbot system in detail. It also explains the biomechanics in-volved in this system [15,50,51].
