# Supplementary material for: Bolstering Cognitive and Locomotor Function in Post-Stroke Dementia Using Human–Robotic Interactive Gait Training
Source: J Clin Med. 2023 Aug 31;12(17):5661. doi: 10.3390/jcm12175661 (PMC10488393; doi:10.3390/jcm12175661)
Supplement: Supplementary file 1 [file jcm-12-05661-s001.zip › Supplementary File S1.pdf]

Supplementary File S1. Conventional physiotherapy

Neurodevelopmental techniques (NDT) using Bobath stress exercises tend to normalize muscle tone and prevent excessive spasticity through special reflex-inhibiting postures and movements. The interventions were grouped into three sessions according to the treatment goal, preparation, facilitated movements, gait-specific activities, and functional activities.

Sessions	Content
Preparation (Normalize muscle tone)	Normalization of muscle tone by proximal mobilization of the lower and upper extremities. Realignment of joints and muscles by specific rotatory mobilization of the muscles. This refers to a linear stretch in the direction of the muscle fibers while maintaining stability through improved alignment of the appropriate joints.<br>1. Hip, knee, and ankle joint mobilizations<br>2. Wrist, elbow, and shoulder joint mobilizations<br>3. Inhibitory mobilizations of lower and upper extremities muscles<br>4. Hand and foot massages
Facilitated movements	The therapist manually guided the performance of normal movement patterns of the limbs, pelvis, and trunk while providing proximal stability to allow for selective movements in the lower and upper extremities. Facilitated movements were sub-grouped according to body segments: proximal (hip, pelvis, and trunk), distal (knee, ankle, and foot), and upper limb.<br>Supine<br>-Logrolling<br>-Supine to side-lying rolling<br>-Selective movement of the upper limb<br>-Selective movement of the hip/knee/ankle/foot<br>-Reaching to the unaffected/affected side<br>-Reaching across the body<br>Side-lying<br>-Holding<br>-Upper trunk rotation<br>-Lower trunk rotation<br>Hook-lying<br>-Holding<br>-Upper trunk rotation<br>-Lower trunk rotation<br>Bridging<br>-Holding

	-Single leg lift<br>-Mobile base of support using a small ball<br>Prone on elbow<br>-Holding<br>-Weight shift<br>-Prone on elbow to quadruped transition<br>Quadruped<br>-Holding<br>-Weight shift<br>-Reaching to the unaffected/affected side<br>Sitting<br>-Holding<br>-Weight shift to unaffected/affected side<br>-Weight shift to anterior/posterior side<br>-Pelvic tilting (anterior/posterior/lateral)<br>-Upper trunk rotation<br>-Reaching to the unaffected/affected side<br>-Movement transitions between sitting to standing<br>Kneeling<br>-Holding<br>-Weight shift to unaffected/affected side<br>-Weight shift to anterior/posterior side<br>-Pelvic tilting (anterior/posterior/lateral)<br>-Upper trunk rotation<br>-Reaching to the unaffected/affected side<br>-Movement transitions between kneeling and heel-sitting position<br>Half-kneeling<br>-Holding<br>-Half-kneeling to standing<br>Standing<br>-Holding<br>-Weight transfer in stride standing/step position<br>-Pelvic tilting (anterior/posterior)<br>-Reaching to the unaffected/affected side<br>-Reaching across the body<br>-Knee flexion/extension with unaffected foot on a step
Gait-specific activities	The different phases of gait or walking were worked on with the assistance of the therapist.<br>Stance phase re-learning

	-Stepping with the unaffected lower limb forward/backward<br>-Stepping with the unaffected lower limb sideways<br>-Stepping with the unaffected lower limb on and off a step<br>Swing phase re-learning<br>-Stepping with the affected lower limb<br>Walking<br>-Forward walking<br>-Backward walking<br>-Side stepping<br>Functional activities<br>-Stair climbing
