# Supplementary material for: Bolstering Cognitive and Locomotor Function in Post-Stroke Dementia Using Human–Robotic Interactive Gait Training
Source: J Clin Med. 2023 Aug 31;12(17):5661. doi: 10.3390/jcm12175661 (PMC10488393; doi:10.3390/jcm12175661)
Supplement: Supplementary file 1 [file jcm-12-05661-s001.zip › Supplementary File S2.pdf]

Supplementary File S2. Walkbot human–robotic interactive gait training system

The Walkbot system, which comprises the important biomechanical features of the interlimb ankle-knee-hip joint locomotor control to generate and assist the natural gait pattern, was developed based on the inverted pendulum model [1]. Its biomechanical features include the built-in kinematic and kinetic computing software (P&S Mechanics, Seoul, Korea), which synchronously analyses both kinematic (e.g., angular displacement) and kinetic (e.g., active and resistive ankle–knee–hip joint forces and torques) [1]. Kinematic data were sampled at 36 Hz using a built-in potentiometer in the Walkbot system. Each joint angle data was determined by the following equations in the schematic model (Figure S1):

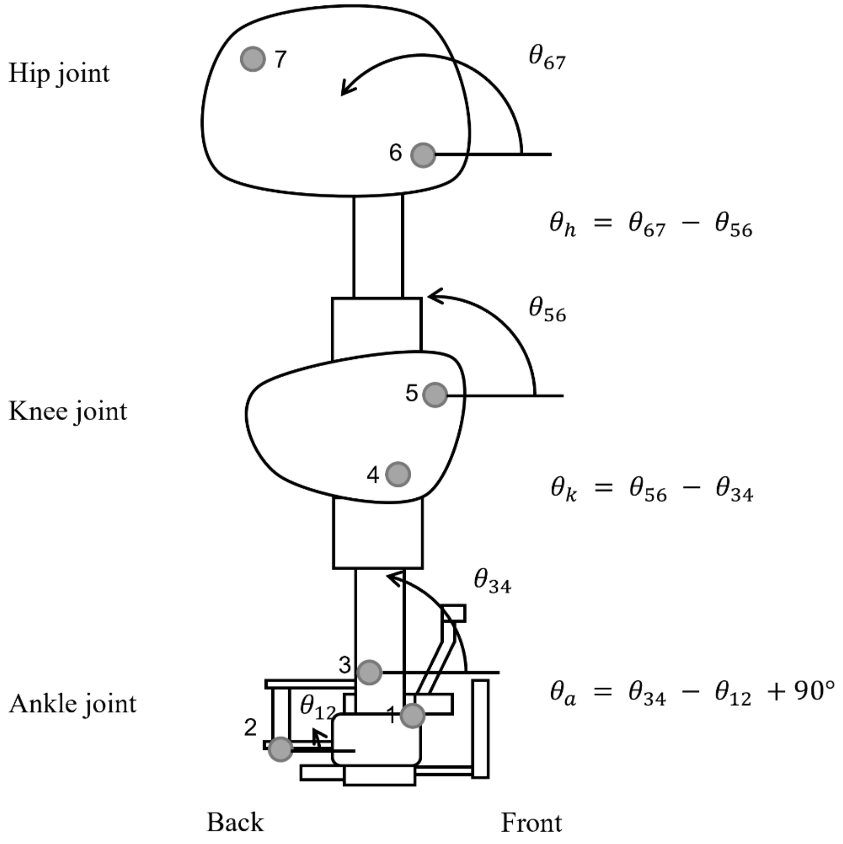

Figure S1. Lower-extremity kinematic joint angle calculation of the Walkbot system. Reproduced with permission from reference [2]. Reproduced with permission from Park C, Oh-Park M, Bialek A, Friel K, Edwards D, You JSH. published by Scientific Reports, 2021.

Kinetic software enables measurement of the forces and torques of the ankle–knee–hip joint related to walking and provides real-time biomechanical feedback to maximize the human–robotic interactive gait training. Based on the kinematic and anthropometric data, the force data were automatically computed and converted to joint torques. All ankle–knee–hip joint torque data were acquired by servomotors mounted in the Walkbot robotic system and used to accurately generate the most natural gait pattern [3] using each

patient's anthropometric data. The specific scheme of the position-based impedance control algorithm is illustrated in Figure S2.

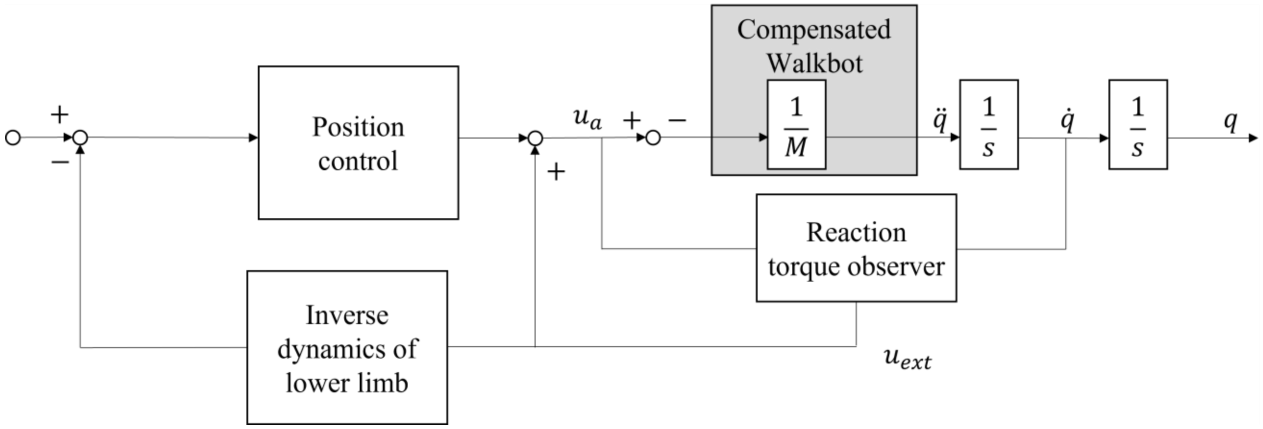

Figure S2. Scheme of the position-based impedance control system. Reproduced with permission from reference [2]. Reproduced with permission from Park C, Oh-Park M, Bialek A, Friel K, Edwards D, You JSH. published by Scientific Reports, 2021.

The force equation is formulated as:

$$u_{ext} = \frac{g}{s+g}\left(u_a + g\hat{F}(q)\dot{q} + \hat{F}(q)\dot{q}\right) - g\hat{F}(q)\dot{q}$$

where u_{ext} represents the estimated external torque from the reaction torque observer, $\frac{g}{s+g}$ is a lowpass filter, and g is its cutoff frequency.

References

1. Van Tran, Q.; Kim, S.; Lee, K.; Kang, S.; Ryu, J. Force/torque sensorless impedance control for indirect driven robot-aided gait rehabilitation system. In Proceedings of the 2015 IEEE International Conference on Advanced Intelligent Mechatronics (AIM), 2015; pp. 652-657.
2. Park, C.; Oh-Park, M.; Bialek, A.; Friel, K.; Edwards, D.; You, J.S.H. Abnormal synergistic gait mitigation in acute stroke using an innovative ankle–knee–hip interlimb humanoid robot: A preliminary randomized controlled trial. *Scientific Reports* **2021**, *11*, 22823.
3. Winter, D.A. *Biomechanics and motor control of human movement*; John Wiley & Sons: 2009.
